# Diagnostic Accuracy of IgA Anti-Transglutaminase Assessed by Chemiluminescence: A Systematic Review and Meta-Analysis

**DOI:** 10.3390/nu16152427

**Published:** 2024-07-26

**Authors:** Dorina Pjetraj, Alfredo Pulvirenti, Marco Moretti, Simona Gatti, Giulia Naspi Catassi, Carlo Catassi, Elena Lionetti

**Affiliations:** 1 Department of Pediatrics, Marche Polytechnic University, 60123 Ancona, Italy; pjetrajdorina@gmail.com (D.P.); s.gatti@staff.univpm.it (S.G.); c.catassi@staff.univpm.it (C.C.); 2 Bioinformatics Unit, Department of Clinical and Experimental Medicine, University of Catania, 95131 Catania, Italy; alfredo.pulvirenti@unict.it; 3 Department of Laboratory Medicine, AOU delle Marche, 60123 Ancona, Italy; marco.moretti@ospedaliriuniti.marche.it; 4 Pediatric Gastroenterology and Liver Unit, Department of Maternal and Child Health, Sapienza-University of Rome, 00161 Rome, Italy; giulia.catassi@gmail.com; 5 Mucosal Immunology and Biology Research Center, Massachusetts General Hospital-Harvard Medical School, Boston, MA 02114, USA

**Keywords:** celiac disease, anti-tissue transglutaminase antibodies, chemiluminescent immunoassay, CLIA, enzyme-linked immunosorbent assay, ELISA, fluorescence enzyme-linked immunoassay, FEIA

## Abstract

A new chemiluminescence immunoassay method (CLIA) for detecting IgA anti-transglutaminase (atTG IgA) in celiac disease (CD) has prompted inquiries into its diagnostic performance. We conducted a systematic review and meta-analysis comparing CLIA with traditional enzyme-linked immunosorbent assay (ELISA) and fluorescence enzyme immunoassay (FEIA). We searched PubMed, Medline, and Embase databases up to March 2024. The diagnostic references were intestinal biopsy and ESPGHAN guidelines. We calculated the sensitivity and specificity of atTG IgA assessed by CLIA and the odds ratio (OR) between the assays. Eleven articles were eligible for the systematic review and seven for the meta-analysis. Sensitivity and specificity of atTG IgA CLIA-assay were 0.98 (95% CI, 0.95–0.99) and 0.97 (95% CI, 0.94–0.99), respectively. The sensitivity of atTG IgA antibody detection did not significantly vary across the three assay modalities examined (CLIA vs. ELISA OR: 1.08 (95% CI, 0.56–2.11; *p* = 0.8); CLIA vs. FEIA OR: 6.97 (95% CI, 0.60–81.03; *p* = 0.1). The specificity of atTG IgA assessed by FEIA was higher than for CLIA (OR 0.17 (95% CI, 0.05–0.62); *p* < 0.007). According to the systematic review, normalization of atTG IgA levels in CD patients following a gluten-free diet was delayed when using CLIA compared to ELISA and FEIA methods. Conflicting findings were reported on the antibody threshold to use in order to avoid biopsy confirmation.

## 1. Introduction

Celiac disease (CD) is an autoimmune enteropathy triggered by gluten ingestion in a subgroup of genetically susceptible subjects positive for HLA-DQ2 and/or HLA-DQ8 [1,2]. Prevalence in the general population is variable, with an average of about 1% and an increasing trend in many countries [3,4,5]. Clinical presentation of CD is highly variable and ranges from classical manifestations, with gastrointestinal and extraintestinal symptoms, to silent forms [6,7]. The current therapy for CD consists of a gluten-free diet (GFD), which is the only approach known to resolve symptoms, allow mucosal healing, and prevent long-term complications [8,9].

The diagnostic approach to CD has greatly evolved in the last few decades. Although intestinal biopsy, showing the typical picture of villous atrophy with increased intra-epithelial lymphocyte count, is still regarded as the gold standard for CD diagnosis, the role of antibody markers has progressively gained a crucial role not only in diagnosis but also in the follow-up of patients affected by this disease. Among the CD markers that have been identified over the years, only three are currently used in clinical practice, i.e., anti-tissue transglutaminase (atTG) IgA, anti-endomysial (EmA) IgA, and anti-deamidated gliadin (DGP) antibodies IgA and IgG [10].

With regard to atTG IgA, the development of an enzyme-linked immunosorbent assay (ELISA) based on human recombinant tTG has represented a cornerstone in antibody celiac serology [11]. Not only does this essay have high sensitivity (90–98%) and specificity (95–97%), but it was able to overcome the limitations of the indirect immunofluorescence required for the EmA test. atTG IgA is not operator-dependent, can be automated, and does not use material from protected animal species as substrate [12]. Besides the ELISA-based test, new laboratory techniques have emerged in recent years, such as the chemiluminescence immunoassay (CLIA) and the fluorescence enzyme immunoassay (FEIA).

**Figure 1:**
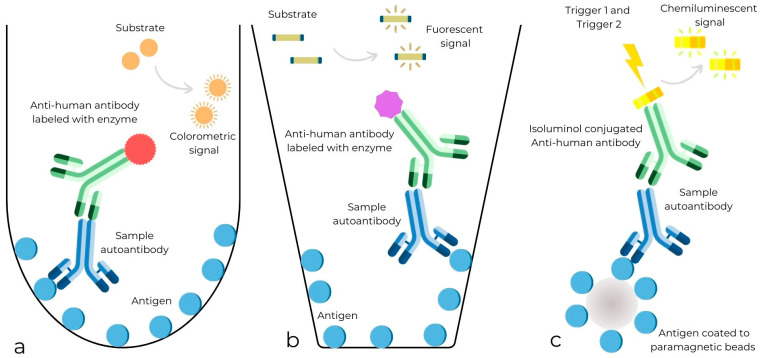
Types of immunoassays. (**a**) Enzyme-linked immunosorbent assay (ELISA): the antigen is non-covalently attached to the plate and the blood sample containing the autoantibody is added. Next, a second antihuman antibody linked to an enzyme is added, followed by a colorimetric substrate. The enzyme catalyzes a reaction of the substrate, creating a color change. The stronger the color, the more target autoantibody is present. (**b**) Fluorescence enzyme immunoassay (FEIA): the antihuman antibodies are labeled with fluorescent dyes that emit fluorescence after the enzymatic reaction. (**c**) Chemiluminescence immunoassay (CLIA): follows similar principles, but the antigen is coated to paramagnetic beads that are incubated with patient samples. After incubation, unbound antibodies are removed by washing, and antihuman IgG isoluminol conjugate is added and binds to immobilized antibodies. Finally, Trigger 1 (oxidizer) and Trigger 2 (catalyst) are injected, and the emerging light is measured as relative luminescence units.

The FEIA technique involves coating the target antigen onto a solid support, which allows it to capture the specific antibodies present in the patient sample. Nonreactive antibodies are then removed through washing, and enzyme-labeled antibodies targeting the captured antibodies are added to form a complex. After an additional incubation step, unbound enzyme-labeled antibodies are washed away, and the bound complex is then incubated with a reagent (developing agent) that undergoes an enzymatic reaction to produce a fluorescent signal. After a defined incubation time, the enzymatic reaction is stopped (stop solution reagent) and the fluorescence is measured with a fluorescence detector. The concentration of antibodies in the patient sample is determined using a standardized calibration curve, thereby producing a quantitative result in addition to negative, equivocal, or positive classifications. Similarly, in the ELISA assay, the enzyme labeled to the antihuman immunoglobulin catalyzes a reaction of the substrate, creating a color change. The stronger the color signal, the more target antigen is present. In the chemiluminescence immunoassay the antigen is coated to paramagnetic beads under conditions that preserve the antigen in its reactive state. The beads are then incubated with patient samples, and after a specific time of incubation, unbound antibodies are removed by washing. Antihuman IgG isoluminol conjugate or acridinium esters (Tracer) are added and bind immobilized antibodies. After another incubation period, unbound Tracer is removed by washing. Finally, Trigger 1 (oxidizer) and Trigger 2 (catalyst) are injected and emerging light is measured as relative luminescence units (Figure 1) [13,14,15,16].

CLIA has become very popular in the last few years, as it enables large-scale testing at a high throughput and is completely automatized. These advantages should be taken into account due to the increasing requests for serology testing [17].

In 2020 the revised guidelines of the European Society for Paediatric Gastroenterology, Hepatology and Nutrition (ESPGHAN) established that CD could be diagnosed in both symptomatic and asymptomatic children by high atTG IgA titers (>10 times the cutoff) and verified by EmA positivity, with no need to obtain duodenal biopsy or HLA typing in these cases. These guidelines were based on 49 pediatric datasets, in which the most used immunoassays were conventional ELISA and FEIA, while only minimal data originated from chemiluminescence-based tests [18].

In this paper we evaluate the performance of the CD-specific antibody atTG IgA by a head-to-head comparison between the chemiluminescence immunoassay (CLIA) and the traditional ELISA and fluorescence enzyme immunoassay (FEIA) based on a meta-analysis and systematic review of available studies.

## 2. Materials and Methods

### 2.1. Protocol

Prior to conducting the review and meta-analysis, we established a comprehensive protocol. This protocol encompassed the eligibility criteria, search strategies, study selection criteria, data extraction methods, and approaches for assessing study quality and statistical methodology. The protocol development was guided by the PRISMA–DTA (Preferred Reporting Items for Systematic Reviews and Meta-Analyses of Diagnostic Test Accuracy) guidelines [19]. This systematic review and meta-analysis is registered with PROSPERO International prospective register of systematic review (ID CRD42024563519).

### 2.2. Eligibility Criteria

The review considered studies of various designs, including comparative prospective, prospective cohort, and retrospective case–control studies, as well as case reports. However, for the meta-analysis, case reports were excluded. Only peer-reviewed, English-language publications were included, regardless of the primary outcomes, publication date, or status. The study population comprised both adults and children. The reference standard for celiac disease diagnosis was either intestinal biopsy or the ESPGHAN guidelines [18]. The primary outcome measure was the diagnostic test accuracy of anti-tissue transglutaminase (atTG) antibodies measured using chemiluminescence immunoassay. The meta-analysis included only comparative primary studies (atTG measured with CLIA and ELISA or FEIA), while comparative primary studies with missing data were included in the review alone. Additionally, studies that followed up a cohort of patients and determined atTG IgA levels during a gluten-free diet were included only in the review.

### 2.3. Information Sources and Search

A comprehensive electronic literature search was conducted across the PubMed and Medline databases, spanning from inception to March 2024. The search strategy involved combining relevant terms for anti-transglutaminase IgA (atTG IgA) and keywords for CD: “(celiac disease [MeSH Terms]) AND (chemiluminescence [MeSH Terms])”. The search protocol adhered to the PRISMA-S guidelines [20]. Additionally, reference lists of all eligible articles were manually scanned, and input was sought from subject matter experts to identify any further relevant records.

### 2.4. Study Selection

The screening, eligibility assessment, and inclusion of studies in the review and meta-analysis were conducted independently by two reviewers following a standardized, unblinded approach without masking authors or institutions. Any discrepancies between the reviewers (D.P. and E.L.) were resolved through mutual agreement.

### 2.5. Data Collection Process

The research team created a data extraction form, which they pilot tested on a random sample of three included studies, refining the form as needed. One investigator extracted data from the selected studies, while a second investigator verified the extracted information.

### 2.6. Definitions for Data Extraction

A true-positive diagnosis was defined as CD confirmed by either the ESPGHAN guidelines or the reference standard (a grade 2 or 3 lesion on small-bowel biopsy according to the Marsh classification, on a scale of 0–3, with higher scores indicating villous atrophy) [21]. A true-negative diagnosis was considered to be non-CD, as evidenced by either a grade 0 lesion on small-bowel biopsy according to the Marsh classification or negative results for all serological markers tested when biopsy was unavailable. The index tests were deemed positive if the numerical value exceeded the normal value specified by the manufacturer or calculated using receiver-operating characteristic curve (ROC) analysis. The following data were extracted from each included study: (1) study characteristics, (2) participant characteristics, and (3) true positives, true negatives, false positives, and false negatives for atTG IgA assessed by CLIA, ELISA, and FEIA.

### 2.7. Risk of Bias and Applicability

The researchers employed the QUADAS (quality assessment of diagnostic accuracy studies) 2 framework to systematically assess the risk of bias and applicability concerns in the included studies across four key domains: patient selection, index test, reference standard, and participant flow and timing [22]. While an overall quality score was not calculated, the biases and relevance within each domain were classified as “low”, “high”, or “unclear”. Both authors independently evaluated the quality of the studies, and any disagreements were resolved through discussion and consensus.

### 2.8. Diagnostic Accuracy Measures

We used the data from contingency tables to calculate sensitivity and specificity with 95% confidence intervals (CIs) for each study. Forest plots were employed to visually represent the estimates of sensitivity and specificity for the individual studies. In order to assess the sensitivity and specificity of the three index tests, we computed the odds ratio (OR) for each study by comparing: (a) false-negative counts for atTG IgA CLIA versus false negatives for atTG IgA ELISA and atTG IgA FEIA, and (b) false-positive counts for atTG IgA CLIA versus false positives for atTG IgA ELISA and atTG IgA FEIA.

### 2.9. Meta-Analysis

The meta-analysis was conducted using the random-effect model and the metapackage in R software 4.3.3. The Mantel–Haenszel inverse variance method was employed for pooling the data. To assess heterogeneity, the *I*^2^ statistic was calculated, which represents the percentage of total variation across the studies that can be attributed to heterogeneity rather than chance [23].

## 3. Results

### 3.1. Study Selection

**Figure 2:**
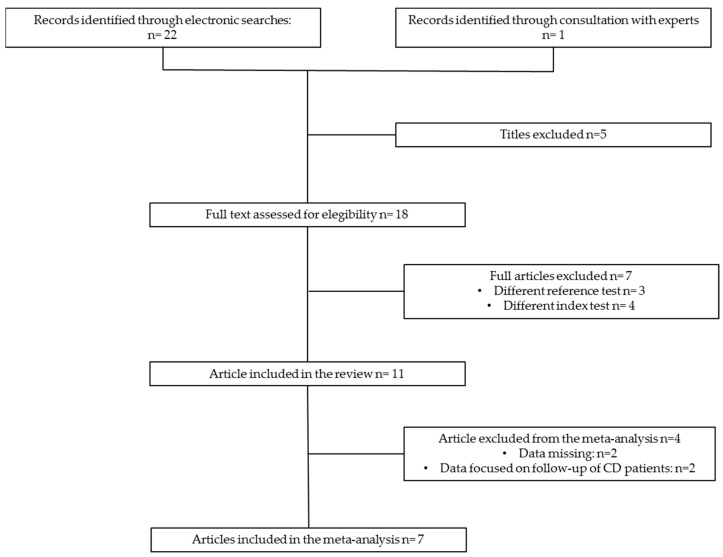
PRISMA flow diagram of study selection.

The flow diagram depicted in Figure 2 provides a visual summary of the literature search process. Initially, 23 articles were identified, of which 18 were deemed potentially relevant and underwent full-text evaluation. Ultimately, 11 articles were deemed eligible and included in the systematic review [17,24,25,26,27,28,29,30,31,32,33], with a subset of 7 of these also incorporated into the meta-analysis [17,24,25,26,27,28,29].

### 3.2. Study Characteristics

Table 1 summarizes the key characteristics of the studies included in the systematic review and meta-analysis [17,24,25,26,27,28,29,30,31,32,33]. Of the seven studies selected for the meta-analysis [17,24,25,26,27,28,29], five were comparative studies that aimed to assess the diagnostic performance CLIA method compared to the FEIA or ELISA methods for the diagnosis of CD in both children and adults [17,24,25,26,27]. These five comparative studies shared a common design: they evaluated a group of patients referred to a tertiary care center for CD screening or suspected CD and were tested for anti-transglutaminase IgA (TtG IgA), and the diagnosis was then confirmed or excluded using the reference standard. They also included a control group of healthy subjects, matched for age and sex, who were negative for serological markers of CD.

Two studies were omitted from the meta-analysis, but were taken into account in the systematic review [30,33]. The first was a prospective comparative study that evaluated the performance of atTG IgA on ELISA and CLIA, although specific data on true-positive and true-negative results required for inclusion in the meta-analysis was not provided [30]. In the second study, performance of atTG IgA on CLIA was retrospectively evaluated compared to anti-endomysium antibody as a reference test; therefore, this study was excluded [33]. Additionally, two more studies examining the normalization trend of atTG IgA levels in CD patients following GFD were incorporated for the purpose of this systematic review [31,32].

The systematic review encompassed a total of 3795 participants, while the meta-analysis involved 2672 participants. Six of the included studies recruited participants from Italy, and all studies enrolled participants seeking care at tertiary health-care facilities.

The included studies exhibited a low risk of bias regarding the index tests. However, the risk of bias related to the reference standard was unclear, as it was not reported whether the pathologist who interpreted the biopsy was consistent throughout the study or whether they were blinded to the serology results. Conversely, the risk of bias pertaining to the flow and timing of the studies was low. No applicability concerns were identified. Notably, none of the articles included in the meta-analysis provided a power calculation to determine the appropriate sample size necessary to address the research question.

### 3.3. Results of Single Studies

**Figure 3:**
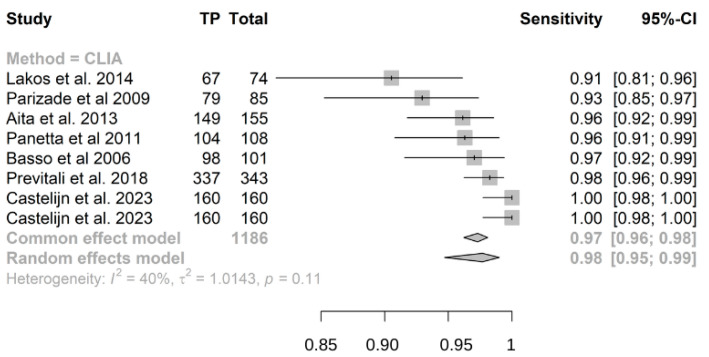
Forest plot for sensitivity of IgA anti-transglutaminase assessed by chemiluminescence (CLIA). True positive (TP); confidence Interval (CI) [17,24,25,26,27,28,29].

**Figure 4:**
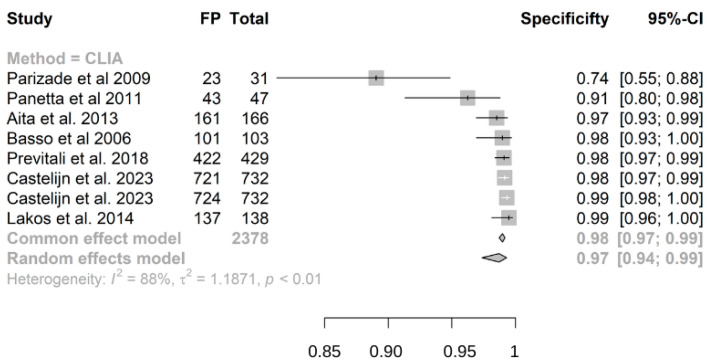
Forest plot for specificity of IgA anti-transglutaminase assessed by chemiluminescence (CLIA). False positive (TP); confidence interval (CI) [17,24,25,26,27,28,29].

Out of 2672 children included in the meta-analysis, 1026 had a confirmed diagnosis of CD by intestinal biopsy or ESPGHAN 2012/2020 guidelines and were therefore considered true positives, while 1646 were true negatives. The summary sensitivity and specificity of atTG IgA on CLIA were 0.98 (95% CI, 0.95–0.99) and 0.97 (95% CI, 0.94–0.99), respectively (Figure 3 and Figure 4). The positive predictive value was determined as 0.96 (95% CI, 0.94–0.97), while the negative predictive value was calculated as 0.99 (95% CI, 0.93–1) (Supplementary Figures S1 and S2).

**Figure 5:**
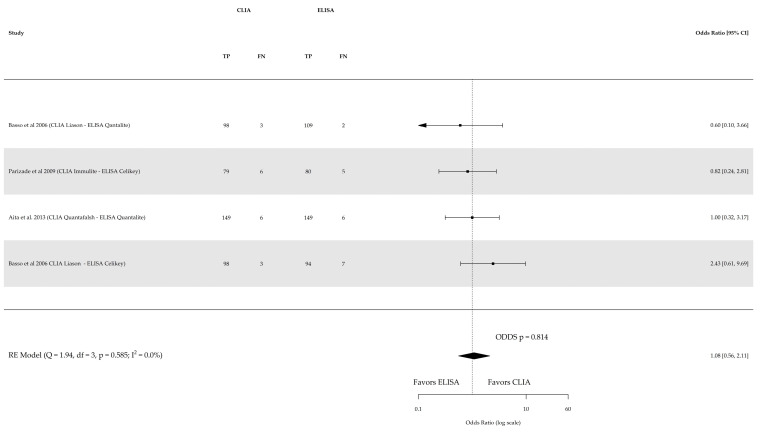
Forest plot of direct comparison of sensitivity of IgA anti-transglutaminase (atTG IgA) assessed by chemiluminescence (CLIA) and enzyme-linked immunosorbent assay (ELISA). Only comparative primary studies reporting the number of total true positives (TPs) and false negatives (FNs) are included. Confidence interval (CI) [17,24,25].

**Figure 6:**
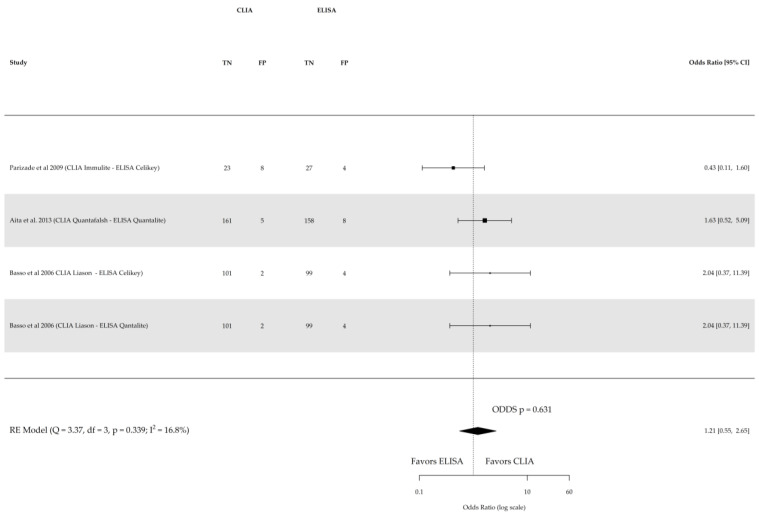
Forest plot of direct comparison of specificity of IgA anti-transglutaminase (atTG IgA) assessed by chemiluminescence (CLIA) and enzyme-linked immunosorbent assay (ELISA). Only comparative primary studies reporting the number of total true negatives (TNs) and false positives (FPs) are included. Confidence interval (CI) [17,24,25].

**Figure 7:**
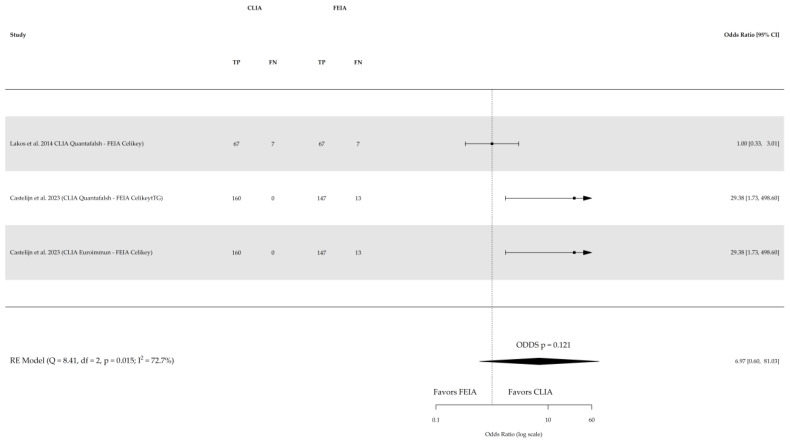
Forest plot of direct comparison of sensitivity of IgA anti-transglutaminase (atTG IgA) assessed by chemiluminescence (CLIA) and fluorescence enzyme immunoassay (FEIA). Only comparative primary studies reporting the number of total true positives (TPs) and false negatives (FNs) are included. Confidence interval (CI) [26,27].

**Figure 8:**
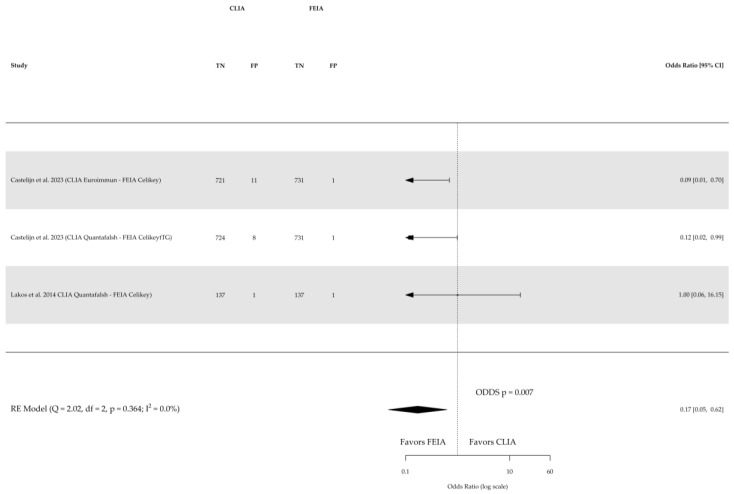
Forest plot of direct comparison of specificity of IgA anti-transglutaminase (atTG IgA) assessed by chemiluminescence (CLIA) and fluorescence enzyme immunoassay (FEIA). Only comparative primary studies reporting the number of total true negatives (TNs) and false positives (FPs) are included. Confidence interval (CI) [26,27].

Direct comparison among the three index tests were conducted based on three studies for sensitivity and specificity of CLIA vs. ELISA (involving 641 subjects) and two studies for sensitivity and specificity of CLIA vs. FEIA (involving 1104 subjects), as depicted in Figure 5, Figure 6, Figure 7 and Figure 8 respectively. No substantial heterogeneity was observed, except in the comparison of sensitivity between CLIA and FEIA. Although the sensitivity of atTG IgA CLIA for CD diagnosis was higher than ELISA and FEIA, the OR for the sensitivity in both cases was not significant (CLIA vs. ELISA OR: 1.08 (95% CI, 0.56–2.11; *p* = 0.8); CLIA vs. FEIA OR: 6.97 (95% CI, 0.60–81.03; *p* = 0.1)), while the OR for specificity was significantly higher for atTG IgA FEIA compared with atTG IgA CLIA (OR: 0.17, 95% CI, 0.05–0.62; *p* = 0.007).

Overall, out of 3795 children included in the systematic review, 1408 had a confirmed diagnosis of CD. Four studies compared atTG IgA assay characteristics for monitoring response to GFD in CD patients [24,25,31,32]. Basso et al. performed two different ELISAs (Celikey and QuantaLite) and a CLIA (Liaison) in 31 CD patients on a GFD (follow-up ranging from 5 to 25 months, median 14.5 months), showing a significant drop in atTG IgA levels after starting the diet, but only the QuantaLite method showed a correlation with gluten withdrawal duration [24]. Another study assessed ELISA versus CLIA performance in monitoring 42 GFD patients over one year, revealing a higher rate of patients presenting positive values atTG IgA assessed with CLIA compared to ELISA, although the earliest and most marked percentage variations were found for CLIA vs. ELISA (*p* = 0.003) [25]. Sansotta et al. focused on children’s follow-up under GFD, finding median time to normalization of atTG IgA of 11.7 months for ELISA and 14.7 months for CLIA (*p* =0.003), with a rate of normalization at 30 months’ follow-up of 86% in the ELISA group and 70% in the CLIA group [31]. Also, Mulder et al. reported that normalization timing of antibody levels is affected by assay type, with CLIA showing a delay compared to FEIA (*p* < 0.001): the time needed for normalization of 50% of the samples was 356 days for the FEIA method and 539 days for the CLIA method [32].

The additional findings from the review pertain to the performance of CLIA in confirming CD diagnosis without biopsy when using ESPGHAN’s recommendation of a 10× upper limit of normal (ULN) cutoff for atTG IgA [18]. Castelijn’s study demonstrated that a larger proportion of patients tested positive with a level ≥10× ULN when assessed with CLIA as opposed to FEIA. Specifically, in the pediatric population, CLIA could potentially have reduced the need for duodenal biopsies in 91–92% of CD children compared to 71% with FEIA [26]. However, Previtali et al.’s study revealed suboptimal PPV (92.1%) when using the 10× ULN cutoff of atTG IgA assessed with CLIA in a pediatric population, but an optimized PPV (99.0%) was achieved at a higher cutoff value of 28× in children. In adults, the same PPV values for Marsh >2 were reached at a 15× cut off (PPV 98%) and 20× cut off (PPV 100%), respectively [28].

## 4. Discussion

The main conclusions drawn from this systematic review and meta-analysis are as follows: (a) the diagnostic accuracy of atTG IgA assessed with CLIA was high (sensitivity 98%, CI 95%: 0.95–0.99; specificity 97%, CI 95%: 0.94–0.99); (b) the sensitivity of atTG IgA assessed by CLIA was slightly higher, but not statistically superior to atTG IgA assessed by ELISA and FEIA, while atTG IgA assessed by FEIA, was superior to CLIA, as far as the specificity is concerned; (c) atTG IgA assessed by CLIA decreased more slowly than ELISA and FEIA in CD patients following GFD; (d) there were conflicting data regarding the accuracy in terms of positive predictive values of atTG IgA assessed by CLIA in confirming CD diagnosis when values are 10 times the cutoff.

This slightly higher sensitivity demonstrated by CLIA may be due to the analytical characteristic of the method, as the reaction kinetics in chemiluminescence may be enhanced by the acridinium ester, which is a chemiluminescent reagent that independently emits light in the presence of triggers without the participation of an enzyme. Moreover, the labeled proteins have a minimal influence on acridinium ester-mediated luminescence intensity. Additionally, the biofunctionalized surface of paramagnetic beads provides a significantly higher surface area compared to a microplate and can immobilize a large amount of antigen or antibody, which often results in high analytical sensitivity and a wider detection range [15,16,24].

Physicians have to take in account that there is a discrepancy in the definition and application of the ULN for atTG IgA levels. While the ULN is typically based on a percentile in a healthy reference population, the ESPGHAN guidelines define it using a cutoff derived from an ROC analysis of CD patients and controls. Furthermore, in the case of an equivocal range, various laboratories and studies may reference the lower or higher end of the range, which can influence clinical interpretation, particularly when atTG IgA levels are moderately elevated. In Catelijn’s study, the cutoff was set according to the manufacturer’s recommendations, and the upper value of the equivocal range was used (7–10 U/mL). Interestingly, ROC analysis suggested an optimal FEIA cutoff of 6.5 U/mL, which is close to the manufacturer’s lower equivocal value. Utilizing this lower cutoff improved sensitivity, especially among adult patients with lower atTG IgA levels compared to pediatric cases [26].

In the context of monitoring patients on a GFD, the data indicate varying timelines for the normalization of serology, depending on the specific assay employed. In some cases, it typically takes over 6 months, but in other instances, there may be ongoing antibody positivity even after 24 months. Additionally, normalization of atTG IgA levels is delayed in children tested using CLIA compared to ELISA and FEIA. To address this concern, it seems prudent to recommend consistent use of the same assay at diagnosis as a surrogate marker for improvement/healing of the small-bowel mucosa in addition to emphasizing the importance of establishing a reliable atTG IgA-level trend during follow-up of patients with CD, in agreement with the latest ESPGHAN position paper [34].

Conflicting findings have been reported regarding the determination of the antibody threshold in cases where biopsy confirmation may be avoided, as indicated by the ESPGHAN guidelines [18]. Aita et al. demonstrated a PPV of 100% for CLIA-measured tTGs using an 10-fold upper limit, but this result was not supported by Previtali et al., who proposed a 28-fold upper limit [25,28]. Additionally, Parizade et al. found lower sensitivity for CLIA compared to ELISA (93.7% vs. 95.2%) when high atTG IgA levels (10× the manufacturer cutoff) are positively associated with Marsh grading [17].This means that more investigation is needed before extending ESPGHAN’s recommendation of greater than 10 times the ULN cutoff for the routine use of chemiluminescence.

To our knowledge, this is the first study to systematically review and meta-analyze the available evidence on the diagnostic accuracy of atTG IgA assessed by CLIA in comparison with ELISA and FEIA. A previous meta-analysis, which included studies published between 2006 and 2015, did not find significant differences in the performance of tTG-IgA assays, but that study only included ELISA or FEIA and did not evaluate the CLIA approach [35].

A key limitation of our review and meta-analysis is the paucity of studies that have primarily compared the diagnostic performance of anti-transglutaminase IgA through alternative assay methods. Additionally, none of the included studies reported conducting a power analysis to determine the appropriate sample size required to address the research question effectively.

## 5. Conclusions

In conclusion, this systematic review and meta-analysis found no statistically significant differences in the diagnostic sensitivity of atTG IgA antibody assessment among the three assay methods (CLIA, ELISA, and FEIA) evaluated, while the specificity of atTG IgA assessed by FEIA was higher than CLIA. Moreover, the review identified conflicting findings regarding the appropriate antibody cutoff value of 10 times the ULN to forgo biopsy confirmation, with reported positive predictive values ranging widely from 92.1% to 100% [17,25,26,28]. Further prospective studies specifically designed to evaluate this issue are needed. Our findings suggest a possible revision of the ESPGHAN guidelines in order to standardize atTG IgA assays, and therefore minimize the risk of misdiagnosis.

## Figures and Tables

**Table 1:** Characteristics of included studies.

References	Study Type and Location	Population	Gender (% Female)	Age Range or Median Age	Reference Test	Index Test	Cutoff Values	True Positive	True Negative
Basso et al. 2006 [24]	Retrospective Italy	204 children: 101 pediatric CD, 103 pediatric controls +31 CD on (GFD).	61	1–15 years	Intestinal biopsy	ELISA (Celikey tTG IgA)	(ROC) 4.4 U/mL	94/101	99/103
						ELISA (Quantalite tTG IgA)	(ROC) 48.0 U	109/111	99/103
						CLIA (Liaison tTG IgA)	(ROC) 16.9 AU/mL	98/101	101/103
Aita et al. 2013 [25]	Retrospective Italy	321 pediatric participants: 155 CD, 166 controls, +42 CD on (GFD)	59	6 years in CD subjects 10 years in controls	Intestinal biopsy	ELISA (Quantalite tTG IgA)	(ROC) 20 U	149/155	158/166
						CLIA (Quantaflash tTG IgA)	(ROC) 16 U	149/155	161/166
Castelijn et al. 2023 [26]	Retrospective Netherlands	892 participants: 160 CD patients (95 adults, 65 children) 732 controls (479 adults, 253 children)	R	18–85 years in adult CD and 0–16 years in pediatric CD	ESPGHAN 2012 guidelines	FEIA (Celikey tTG IgA)	(Ma) 10 U/mL	147/160	731/732
						CLIA (Quantaflash tTG IgA)	(Ma) 20 U/mL	160/160	724/732
						CLIA tTG IgA (Euroimmun)	(Ma) 10 U/mL	160/160	721/732
Lakos et al. 2014 [27]	Retrospective USA	212 adults: 74 CD and 138 controls	76	19–83 years	Intestinal biopsy	FEIA (Celikey tTG IgA)	(Ma) 7–10 U/mL	67/74	137/138
						CLIA (Quantaflash tTG IgA)	(Ma) 20 U/mL	67/74	137/138
Previtali et al. 2018 [28]	Retrospective Italy	772 participants: adults and children (343 CD and 429 controls)	62	43 years in adults 9 years in children	Intestinal biopsy	CLIA (Quantaflash tTG IgA)	(Ma) 20 U/mL	337/343	422/429
Parizade et al. 2009 [17]	Prospective Israel	116 children: 85 CD and 31 controls	55	1–17 years	Intestinal biopsy	CLIA TG IgA (Immulite 2000)	(Ma) 4 U/mL	79/85	23/31
						ELISA (Celikey tTG IgA)	(Ma) 5 U/mL	80/85	27/31
Panetta et al. 2011 [29]	Retrospective Italy	155 children < 2 years: 108 CD 47 controls	75	<2 years	Intestinal biopsy	CLIA (Liason tTG IgA)	(Ma) 8 AU/mL	104/108	43/47
Belei et al. 2020 [30]	Prospective Romania	75 children: 35 CD 40 controls	R	2–18 years	Intestinal biopsy	ELISA (Immulisa tTG IgA)	(Ma) >25 EU/mL	R	R
						CLIA TG IgA (Immulite 2500)	(Ma) >4 U/mL)	R	R
Sansotta et al. 2020 [31]	Retrospective Italy	260 CD children	R	5.1 years in ELISA group 7.7 years in CLIA group	Intestinal biopsy	ELISA (Celikey Varelisa IgA)	(Ma) 2.5 U/mL	R	R
						CLIA (Quantaflash tTG IgA)	(Ma) 20 U/mL	R	R
Mulder et al. 2023 [32]	Retrospective Netherlands	61 participants: 44 CD adults and 17 CD children	72	18–72 years in adults 0–14 years in children	Intestinal biopsy and ESPGHAN guidelines	FEIA (Celikey tTG IgA)	(Ma) 7 U/mL	R	R
						CLIA (Quantaflash tTG IgA)	(Ma) 20 U/mL	R	R
						CLIA (Euroimmun)	(Ma) 10 U/mL	R	R
Daves et al. [33]	Retrospective Italy	727 adults (26 CD patients and 701 controls)	66	27 years	Anti-Endomysium antibody (EmA)	CLIA (Quantaflash tTG IgA)	(Ma) 20 U/mL	26/26	698/701

CD: celiac disease, R: not reported, GFD: gluten-free diet; Ma: manufacturer’s recommended cutoff values, ROC curve recommended cutoff values.

## Data Availability

The authors confirm that the data supporting the findings of this study are available within the article and its Supplementary Materials.

## Supplementary Materials

The following supporting information can be downloaded at https://www.mdpi.com/article/10.3390/nu16152427/s1, Figure S1: Forest plot of positive predictive value (PPV) of atTG IgA assessed by chemiluminescence. Figure S2: Forest plot of negative predictive value (NPV) of atTG IgA assessed by chemiluminescence.
