# Supplementary figures and images for: Diagnostic Accuracy of IgA Anti-Transglutaminase Assessed by Chemiluminescence: A Systematic Review and Meta-Analysis
Source: Nutrients. 2024 Jul 26;16(15):2427. doi: 10.3390/nu16152427 (PMC11314508; doi:10.3390/nu16152427)

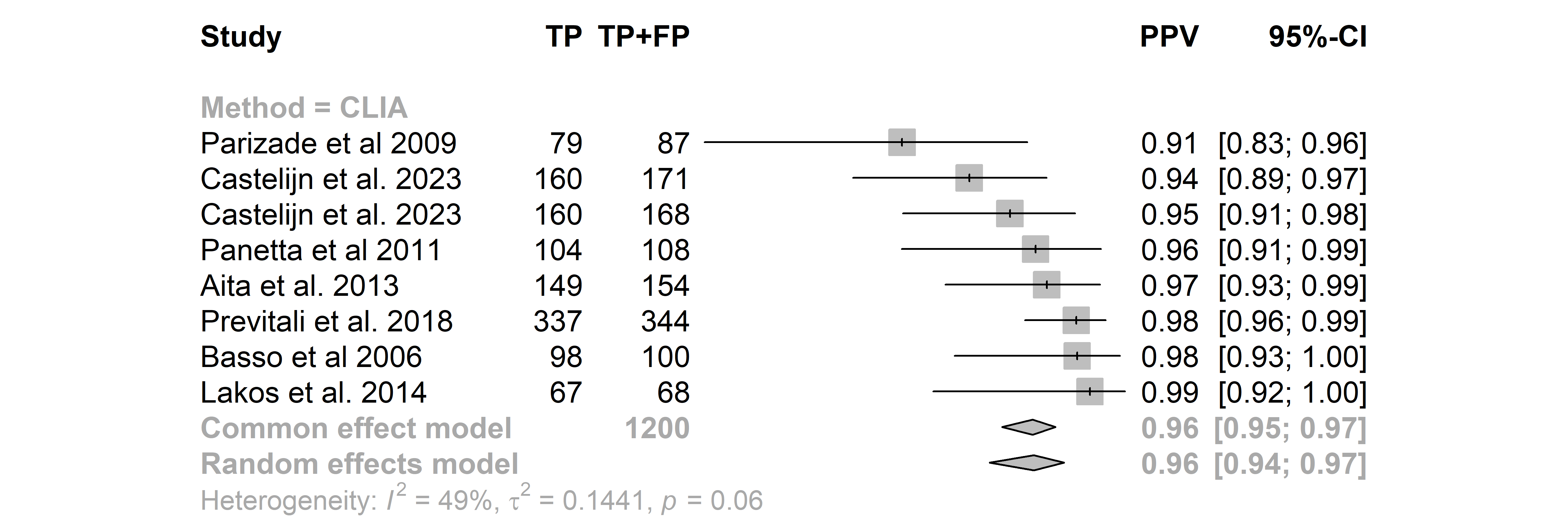

Supplement: Supplementary file 1 [file nutrients-16-02427-s001.zip › Supplementary Figure S1.png]

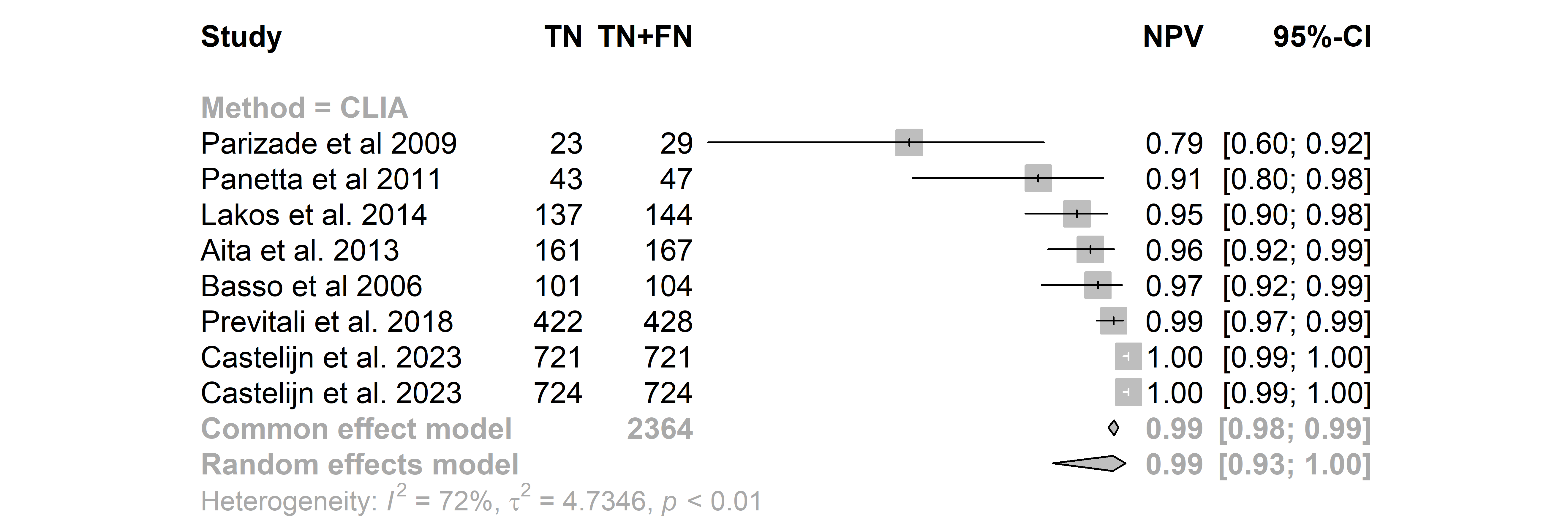

Supplement: Supplementary file 1 [file nutrients-16-02427-s001.zip › Supplementary Figure S2.png]
